# Assessing changes in clusters of wildlife road mortalities after the construction of wildlife mitigation structures

**DOI:** 10.1002/ece3.8053

**Published:** 2021-09-01

**Authors:** Thomas J. Yamashita, Trinity D. Livingston, Kevin W. Ryer, John H. Young, Richard J. Kline

**Affiliations:** ^1^ School of Earth, Environmental, and Marine Sciences University of Texas Rio Grande Valley Port Isabel TX USA; ^2^ Caesar Kleberg Wildlife Research Institute Texas A&M University –Kingsville Kingsville TX USA; ^3^ Environmental Affairs Division Texas Department of Transportation Austin TX USA

**Keywords:** local hot spot analysis, Mann–Kendall test, road ecology, wildlife mitigation structures, wildlife road mortality

## Abstract

Collisions with vehicles can be a major threat to wildlife populations, so wildlife mitigation structures, including exclusionary fencing and wildlife crossings, are often constructed. To assess mitigation structure effectiveness, it is useful to compare wildlife road mortalities (WRMs) before, during, and after mitigation structure construction; however, differences in survey methodologies may make comparisons of counts impractical. Location‐based cluster analyses provide a means to assess how WRM spatial patterns have changed over time. We collected WRM data between 2015 and 2019 on State Highway 100 in Texas, USA. Five wildlife crossings and exclusionary fencing were installed in this area between September 2016 and May 2018 for the endangered ocelot (*Leopardus pardalis*) and other similarly sized mammals. Roads intersecting State Highway 100 were mitigated by gates, wildlife guards, and wing walls. However, these structures may have provided wildlife access to the highway. We combined local hot spot analysis and time series analysis to assess how WRM cluster intensity changed after mitigation structure construction at fine spatial and temporal scales and generalized linear regression to assess how gaps in fencing and land cover were related to WRM cluster intensity in the before, during, and after construction periods. Overall, WRMs/survey day decreased after mitigation structure construction and most hot spots occurred where there were more fence gaps, and, while cluster intensity increased in a few locations, these were not at fence gaps. Cluster intensity of WRMs increased when nearer to fence gaps in naturally vegetated areas, especially forested areas, and decreased nearer to fence gaps in areas with less natural vegetation. We recommend that if fence gaps are necessary in forested areas, less permeable mitigation structures, such as gates, should be used. Local hot spot analysis, coupled with time series and regression techniques, can effectively assess how WRM clustering changes over time.

## Graphical abstract

Wildlife mitigation structures are often constructed to reduce wildlife road mortalities; however, gaps in the fence may reduce the effectiveness of these structures. We assessed how hot spots of wildlife road mortality changed with construction of mitigation structures using emerging hot spot analysis and generalized linear regression. We found that hot spot intensity has not changed after 1 year postconstruction of mitigation structures and that resulting spatial patterns of wildlife road mortalities were not associated with the locations of mitigation structures.

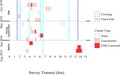

## 1 INTRODUCTION

The distribution of wildlife road mortalities (WRMs) is often affected by species, road, and landscape attributes (Ascensão et al., 2017; Clevenger et al., 2001), and characterizing spatial patterns of WRMs is often beneficial for developing and assessing mitigation measures (Andis et al., 2017). However, counts of WRMs are not always a good measure of clustering (Teixeira et al., 2017), and clustering and counts of WRMs are often associated with different environmental factors (Bíl et al., 2019; Snow et al., 2014). Additionally, long‐term WRM datasets may be affected by variation in detection rates through time due to changes in survey methodology and researcher experience, so examining counts may bias conclusions about how WRM patterns have changed over time. Finally, mitigation structures could cause there to be fewer WRMs along a highway, but because they become more concentrated around gaps in fencing (van der Ree et al., 2015), researchers may draw different conclusions about the effectiveness of mitigation structures depending on whether they examine counts or clustering of WRMs.

Different methods exist to examine how WRM spatial clustering changes through time, including kernel density estimation and time series analyses of clustering algorithms such as hot spot analysis and Moran's I analysis. Kernel density estimation creates a probability surface of a road where hot spots can be identified based on a defined isopleth threshold, while hot spot analysis and Moran's I use location‐based nearest neighbor clustering algorithms to identify where hot spots occur (Anselin, 1995; Getis & Ord, 1992; Snow et al., 2014). While both kernel density estimation and a location‐based approach can be used to identify patterns through time, kernel density estimation is more strongly affected by small sample sizes, such as WRM datasets, potentially causing isolated WRMs to have a strong influence on the probability surface generation causing an overestimation of hot spot locations. While a location‐based approach is also affected by small sample sizes, it is less affected by isolated WRMs. Using a location‐based approach also allows one to explicitly examine how the intensity and distribution of WRM clusters changes through time using time series analysis such as the Mann–Kendall test (Getis & Ord, 1992; Harris et al., 2017).

Local hot spot analysis measures whether block values are high relative to surrounding blocks (Getis & Ord, 1992), while local Moran's I analysis measures whether block values are high relative to all other blocks (Anselin, 1995). Both measures use a weighting factor to determine how much influence neighboring blocks have on a particular block. When studying changes in WRMs, researchers are typically interested in how WRMs in particular locations change over time, and local hot spot analysis is better than both Moran's I and kernel density estimation at identifying how this pattern changes (Getis & Ord, 1992).

Using local hot spot analysis to identify WRM clusters also allows one to examine how the intensity of a cluster is affected by environmental factors and how this relationship changes through time. Factors that influence the distribution of WRM clusters include variation in land cover and land use (Ascensão et al., 2017; Caceres, 2011), highway characteristics (Clevenger et al., 2003; Grilo et al., 2015), and the presence of wildlife mitigation structures, especially exclusionary fencing (Cserkész et al., 2013). Fencing restricts access to roadways to narrow gaps at road intersections and private drives which can decrease the overall number of WRMs on the highway (Forman et al., 2003); however, it could increase the intensity of WRM clusters near these locations by funneling animals toward gaps in the fences (Cserkész et al., 2013). The potential for funneling is often a concern in wildlife mitigation structure construction (Huijser et al., 2016), so gaps are often mitigated by various structures including gates, wildlife guards, and wing walls. These structures are not 100% effective at keeping wildlife off roads, and WRMs may still result (Allen et al., 2013; van der Ree et al., 2015). Therefore, examining how fence gaps influence the intensity of WRM clusters may be important in determining how wildlife mitigation structures affect WRMs.

We used local hot spot analysis to assess how WRM clusters changed through time with the construction of wildlife mitigation structures on State Highway 100 (SH100) in Cameron County, Texas, USA. We examined how the intensity of WRM clusters changed with mitigation structure construction at a fine temporal scale and how factors influencing WRM cluster intensity changed from before construction to after construction of wildlife mitigation structures. We expected to see fewer WRM clusters in the after construction period than the before‐ and during construction periods coupled with increased cluster intensity due to limited access to the road area. We also expected that the intensity of WRM clusters would decrease with increased distance to wildlife mitigation structures in the after construction period only.

## 2 METHODS

### 2.1 Study area

**FIGURE 1:**
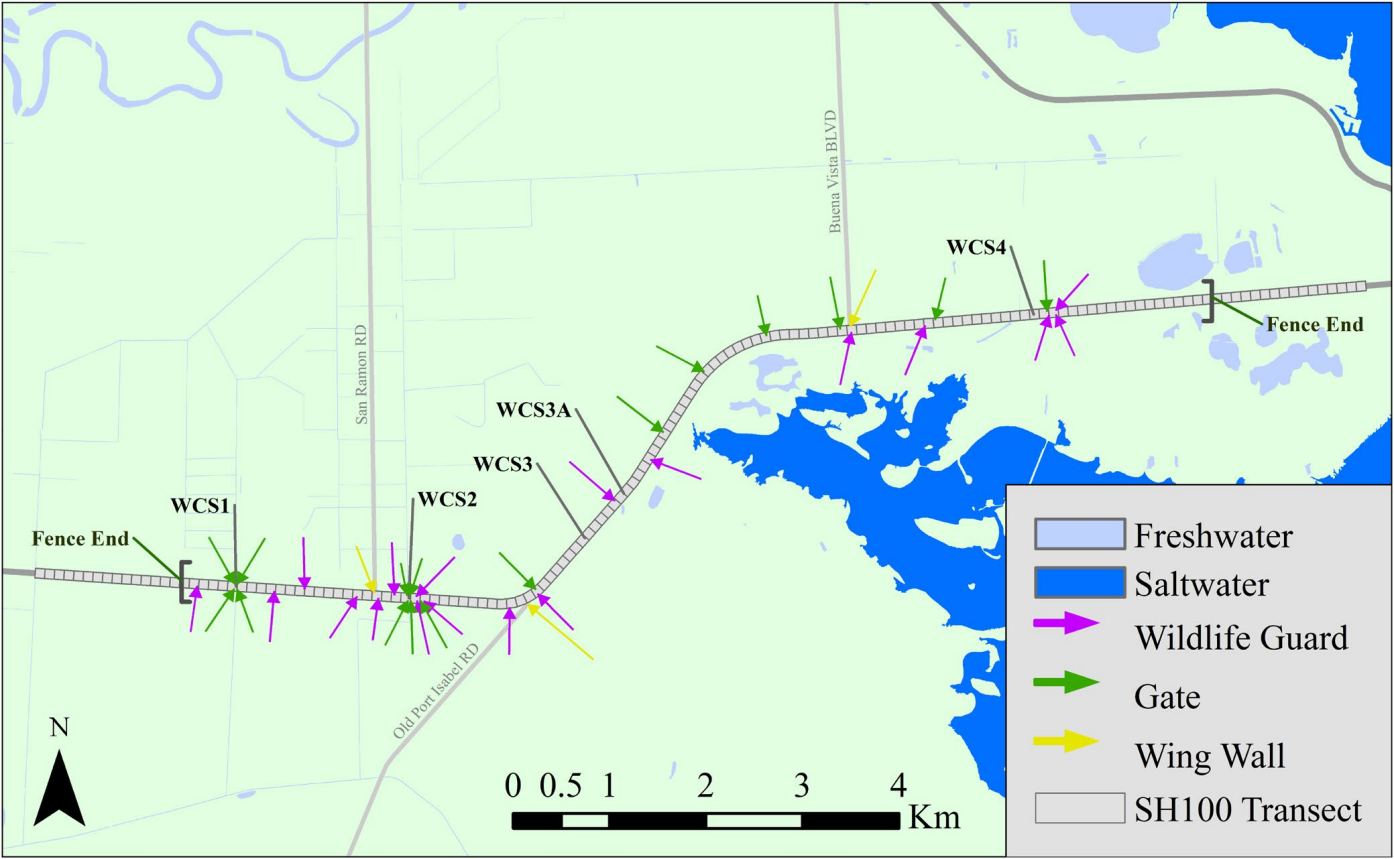
Map of the wildlife mitigation area on State Highway 100 showing the three types of fence gaps: gates, wildlife guards, and wing walls. The wildlife road mortality survey transect is divided into 151 100‐m road segments

The study area was a 15‐km section of SH100 in Cameron County, Texas, USA, between the towns of Laguna Vista and Los Fresnos (Figure 1). The highway is a four‐lane road with a concrete traffic barrier median. This section of SH100 had a speed limit of 105 kmh and an average annual daily traffic of between 7,000 and 9,000 vehicles (Texas Department of Transportation, 2019). Within the survey transect, wildlife mitigation structures were built between September 2016 and May 2018. The survey transect included the entire mitigation area and 1.5 km on either side of it.

Mitigation structures built included 11.9 km of exclusionary fencing along the entire mitigation area, five wildlife underpasses, 18 wildlife guards, three wing walls, and 16 gates. The mitigation structures were designed to prevent ocelots (*Leopardus pardalis*), bobcats (*Lynx rufus*), and other medium to large mammals from accessing the road, while still providing connectivity across the highway (Environmental Affairs Division, 2015). The fencing material was 5.1 cm wide black plastic‐coated chain‐link, 1.8 m tall, and was buried 30.5 cm into the ground along most of the fence line. In areas where burial was not possible, the fence was secured to the ground away from the highway.

Cameron County is characterized by hot summers with an average daily temperature in August of 29.6℃ and mild winters with an average daily temperature in January of 16.2℃ (National Weather Service, 2020). The area receives an average of 69.7 cm of rain per year, and most rainfall occurs during occasional tropical storms between June and October. The primary vegetation types in the study area were cordgrass prairie, salt marsh, and thornscrub forest (Elliott et al., 2014).

### 2.2 Wildlife road mortality surveys

Wildlife road mortality surveys were conducted by vehicle before, during, and after the construction of the mitigation structures on SH100. The survey transects included the full mitigation area as well as a 1.5 km buffer on both sides. Survey frequency, speed, and marking differed in the three construction periods (Table 1), resulting in variation in the total number of surveys conducted among periods. In all survey periods, two people conducted the survey, mammals and reptiles were recorded, and the GPS location of each mortality was recorded. In the before construction period (August 2015–August 2016), the vehicle was driven around 40 kmh and two surveys were conducted per month (total 30 surveys). In the during construction period (September 2016–May 2018), the vehicle was driven 48–64 kmh and two surveys were conducted per week (total 127 surveys). In the after construction period (June 2018–September 2019), the vehicle was driven 48–64 kmh and one survey was conducted per week (total 67 surveys). The switch to one survey per week was due to a previous study on SH100 that showed that most carcasses remained identifiable for at least a week (Livingston, 2019). Previous studies have recommended slower speeds for vehicle‐based surveys to accurately detect all road mortalities than what were used in this study (Collinson et al., 2014; Santos et al., 2011). However, because SH100 is a high‐speed, high traffic road, it would have been unsafe for the researchers to drive any slower.

**TABLE 1:** Comparison of wildlife road mortality survey methodologies among construction periods on State Highway 100, Cameron County, Texas

	Before	During	After
Surveys/month	2	8	4
Time period	August 2015–August 2016	September 2016–May 2018	June 2018–September 2019
Vehicle speed	40 kmh	48–64 kmh	48–64 kmh
People/Vehicle	2	2	2
Coordinates	GPS	GPS	GPS
Photograph	No	Yes	Yes
Carcass removal	Marked but not removed	Unmarked and not removed	Unmarked and not removed
Taxa recorded	Mammalia, Reptilia	All	All

Note

Surveys per month are the approximate number of wildlife road mortality surveys conducted per month, time period is the dates that surveys were being conducted, and carcass removal indicates if carcasses were marked or moved by surveyors.

Only those species for which fencing provided a barrier to movement were used in analyses to assess how fencing changed WRM patterns. These included all mammals larger than rodents as well as turtles and tortoises (Table 2). All analyzed taxa were recorded during all three survey periods. Snakes, amphibians, birds, and small mammals were not included in analyses; see Appendix A for a complete list of species found during WRM surveys.

**TABLE 2:** Total number of wildlife road mortalities by class before, during, and after construction of wildlife mitigation structures on State Highway 100, Cameron County, Texas. For a complete breakdown of wildlife road mortalities by species and time period, see Appendix A

Group	Class	Before	During	After	Total mortalities
Months of data		11	20	16	–
Target species	Mammalia	89	140	114	343
	Reptilia	28	4	16	48
	Total	117	144	130	391
Nontarget species	Aves	5^a^	50	101	156
	Mammalia	36	12	25	73
	Reptilia	67	19	40	126
	Malacostraca	0	0	6	6
	Unknown	1	0	1	2
	Total	109	81	186	376
Grand total		226	225	316	767

^a^ While birds were not surveyed in the before construction period, we did record a few, primarily in the surveys at the end of the period.

### 2.3 Land cover classification

To identify land cover types around SH100, we created a classified vegetation map using an image from the National Agriculture Imaging Program (NAIP; year taken: 2016). We classified the image into 10 classes using the Interactive Supervised Classification Tool in ArcMap 10.6 (ESRI, 2017): trees, shrubs, cactus, cordgrass, open, bare, paved road, dirt road, water, and bahia. Classification was confirmed by visual inspection of the map. These classes were simplified to three major land cover types: forested (trees, bahia), shrub (shrubs, cactus), and open (open, bare, paved road, dirt road). The water class was excluded because water was identified using a different method, described below.

We identified permanent sources of fresh and saltwater using the National Wetlands Inventory (U.S. Fish & Wildlife Service, 2018). Saltwater areas were identified as all locations that had the saltwater, tidal regime subgroup and included the subtidal, irregularly exposed, regularly flooded, and irregularly flooded water regimes. Permanent freshwater areas were those that were classified into the nontidal regime subgroup and had the permanently flooded, intermittently exposed, or semipermanently flooded water regimes. In addition to these sources of permanent freshwater, the drainage canals around SH100 were included because they had flowing water throughout most of the year. We extracted linear water features from the National Wetlands Inventory that had the excavated tag and created a 3‐m buffer around these using ArcMap 10.6 to capture the full width of the canals. The locations and sizes of the drainage canals were confirmed using published maps available from the Cameron County Drainage District (Cameron County Drainage District #1, 2010).

To identify agricultural and developed areas, we manually digitized an ESRI orthoimage (year taken: 2018). Developed areas included all buildings, wind turbines power stations, utility towers, and roads. We manually digitized buildings and used the TxDOT roads database (Texas Department of Transportation, 2020) to identify most roads in the study area. We digitized any other roads visible in the orthoimage manually. Most of these were new roads associated with construction of the San Roman Wind Farm and new housing developments. We created a 20‐m buffer around all paved roads to encompass the full road area as well as the right of way and a 10‐m buffer around all dirt roads. We confirmed agricultural and developed areas using orthoimagery taken in 2013 and 2016, the Cameron County Parcel information from 2019 (Cameron CAD, 2020), and by visits to sites.

We combined the water, agriculture, and developed layers with the classified vegetation map using the reclassify and raster calculator tools in ArcMap 10.6 producing a final map with seven classes: saltwater, freshwater, developed, agriculture, forested, shrub, and open.

### 2.4 Changes in wildlife road mortalities through time

We assessed changes in WRM cluster intensity through time by coupling local hot spot analysis and a time series analysis. We divided our WRM location dataset into space time blocks that were 100‐m × 4 months. We used 100‐m space blocks because fence gaps are highly localized features, and this block size best represented the spatial relationship between blocks and gaps. We tried smaller and larger block sizes, but the 100‐m block performed the best. We used 4‐month time blocks (June–September, October–January, February–May) because this block size fits both the construction periods and seasonal rainfall patterns and movement of wildlife in South Texas.

To assess changes in clustering through time, we ran a local hot spot analysis using ArcMap 10.6 on each time block (4‐month period) to determine the intensity of WRM clustering at each space block (100‐m segment). The clustering measure we used relies on the relative number of WRMs in a time block so comparison of cluster intensity should not be biased by survey frequency and vehicle speed, assuming that any detection biases associated with these factors are consistent along the entire survey transect. Next, we ran the Mann–Kendall test using the “mk.test” function in the *trend* package in R (Pohlert, 2018) on the z‐score from the hot spot analysis, representing cluster intensity, to determine how clustering at each space block has changed through time. We applied the false discovery rate (FDR) correction for multiple samples and spatial autocorrelation when testing for statistical significance of the local hot spot analyses and Mann–Kendall test using the “p.adjust” function in R (R Development Core Team, 2019). The FDR correction is superior to more conservative corrections, such as the Bonferroni correction, for the identification of spatial and temporal clustering because it is less likely to miss a true cluster without identifying false clusters (Caldas de Castro & Singer, 2006). Because the FDR correction, like most multiple sample corrections, is sensitive to low sample sizes (Caldas de Castro & Singer, 2006), we assessed spatiotemporal trends in WRM clustering visually using both corrected significance and uncorrected significance.

### 2.5 Impact of fence gaps on wildlife road mortality cluster intensity

**FIGURE 2:**
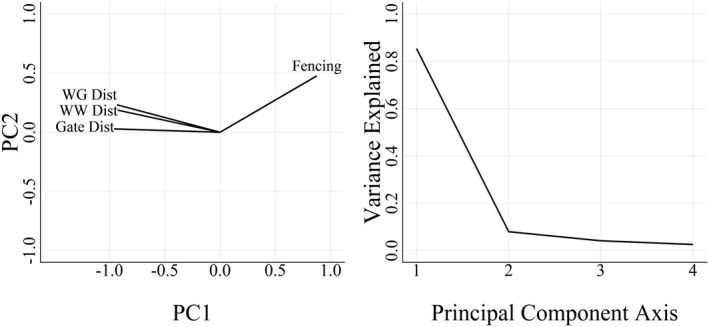
Biplot showing the correlation between the first two principal component axes and distance to wildlife guard (WG Dist), distance to wing wall (WW Dist), distance to gate (Gate Dist), and fence presence (Fencing) from a 100‐m road segment on State Highway 100, Cameron County, Texas (left), and a scree plot showing the proportion of variance explained along each principal components axis (right)

We also tested how the presence of gaps in the fence influenced the intensity of WRM clusters. We were interested in comparing cluster intensity in the three construction periods, instead of time blocks, so we ran local hot spot analysis on each of the three construction periods (before, during, and after) to create a comparable measure of WRM clustering among the three periods. We measured the distance from each space block to three different types of fence gaps (gates, wildlife guards, and wing walls) and recorded whether there was continuous fencing within each space block. In space blocks at the edges of the mitigation area, fencing was determined by whether or not the majority of the block had fencing. Distances to each fence gap type and fence presence were highly correlated to each other (*r* = 0.72–0.88) so we performed a principal components analysis (PCA) using the “prcomp” function in R to develop an index representing distance to fence gaps. The first principal components (PC) axis, hereafter fence gap index, explained 85% of the variation in distance to fence gaps, so it was the only axis used in the regression. Positive values of the first axis represented locations that were closer to gaps and unfenced areas (Figure 2).

To assess how local land cover was related to clustering intensity, we created 100‐m buffers around each space block. We performed this analysis at the local scale because WRM risk has been shown to be associated with the presence of specific habitat features such as freshwater sources, access to roads, or movement corridors (Červinka et al., 2015; Grilo et al., 2016), and we expected that this distance would be small enough to assess these local scale effects. Additionally, at larger spatial scales the influence of fence gaps is overshadowed by larger scale landscape effects such as habitat type. We calculated the proportion of each cover type within the buffer using an iterative version of the tabulate area tool in ArcMap 10.6.

We conducted a generalized linear regression with a Gaussian error distribution to assess how cluster intensity was related to fence gap index, the proportions of forested, shrub, open, agriculture, developed, and freshwater, and the interactions between the fence gap index and land cover variables. No saltwater was located within any of the buffers. We did not include distance to wildlife crossing in the final models because the variable was never significant and did not improve model fit. While road characteristics such as traffic volume, road size and type, and speed limit may also impact WRMs (Clevenger et al., 2001; Grilo et al., 2015), the variations in these characteristics were minor along SH100, so they were excluded.

We used the *MuMIn* package in R to perform AICc model selection and model averaging to model the relationship between cluster intensity and fence gap index and land cover (Barton, 2013; Burnham & Anderson, 2002). The relevant main effects were always included in models containing interactions. Models that were within two ΔAICc values of the best model were used for averaging. We calculated the McFadden pseudo‐*R* ^2^ values for individual models included in the averaged model using the *pscl* package in R (Jackman, 2012).

## 3 RESULTS

### 3.1 Change in wildlife road mortalities through time

**FIGURE 3:**
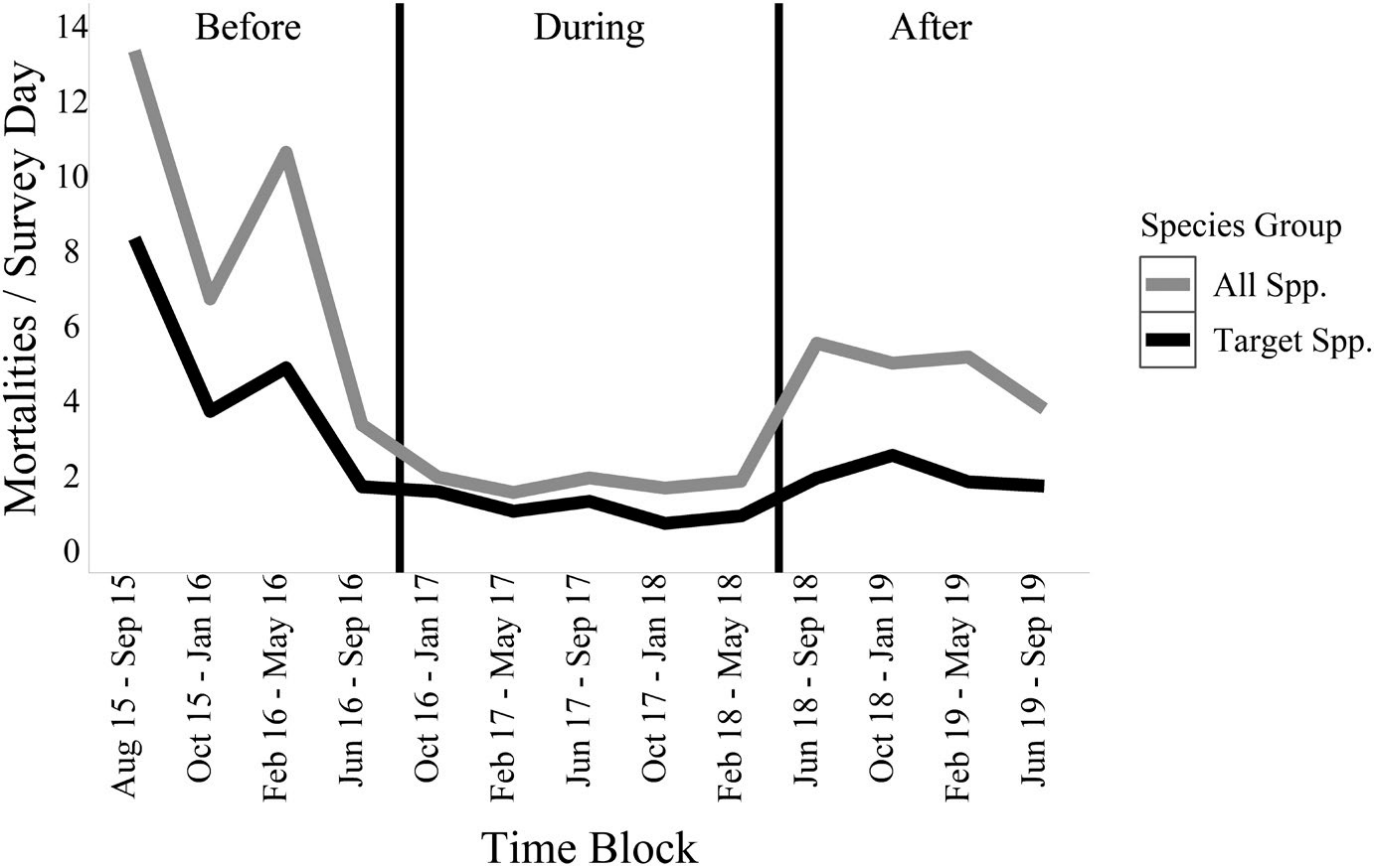
Total number of wildlife road mortalities per time block normalized by number of survey days along State Highway 100, Cameron County, Texas. Wildlife road mortalities shown include target species (mammals larger than rodents, turtles, and tortoises) and all species combined (target plus nontarget species). Vertical lines delineate the periods before, during, and after the construction of wildlife mitigation structures

**FIGURE 4:**
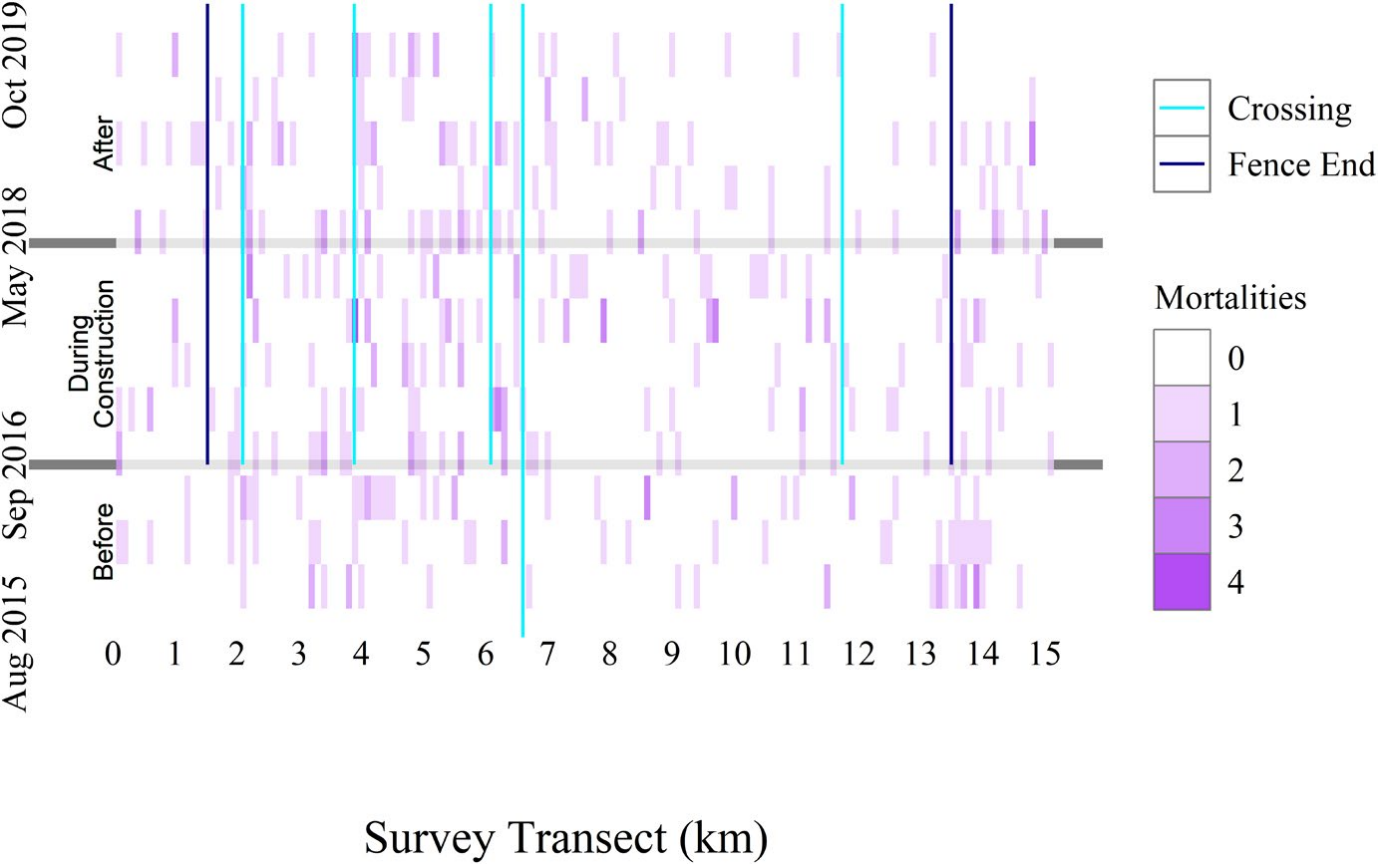
Number of wildlife road mortalities by space time block along State Highway 100 (SH100), Cameron County, Texas. SH100 was divided into 151 100‐m road segments and 13 time blocks, and each block was filled with the number of wildlife road mortalities during that period. The survey transect blocks represent road segments and increase from west to east. To better relate this to the study area map, the approximate locations of wildlife crossings and fence ends are also indicated by vertical lines and the construction periods are indicated by horizontal lines

In total, we surveyed 3,360 km of road and identified 391 target species WRMs (13–44 per time block) and 376 nontarget WRMs (10–60) (Table 2). Most target species WRMs were mammals, with Virginia opossum (*Didelphis virginiana*), eastern cottontail (*Sylvilagus floridanus*), and northern raccoon (*Procyon lotor*) making up the majority of WRMs throughout all time blocks (Appendix A). In the before construction period, there were 5.3 WRMs/survey day, 0.9 WRMs/survey day in the during construction period, and 2.0 WRMs/survey day in the after construction period (Figure 3). There was greater variation in WRMs/survey day in the before construction period when only two surveys were conducted per month than in either of the other periods when more surveys were conducted (Figure 3). Visually, the majority of WRMs occurred on the western side of the survey transect, an area with most of the wildlife crossings and fence gaps (Figure 4).

**FIGURE 5:**
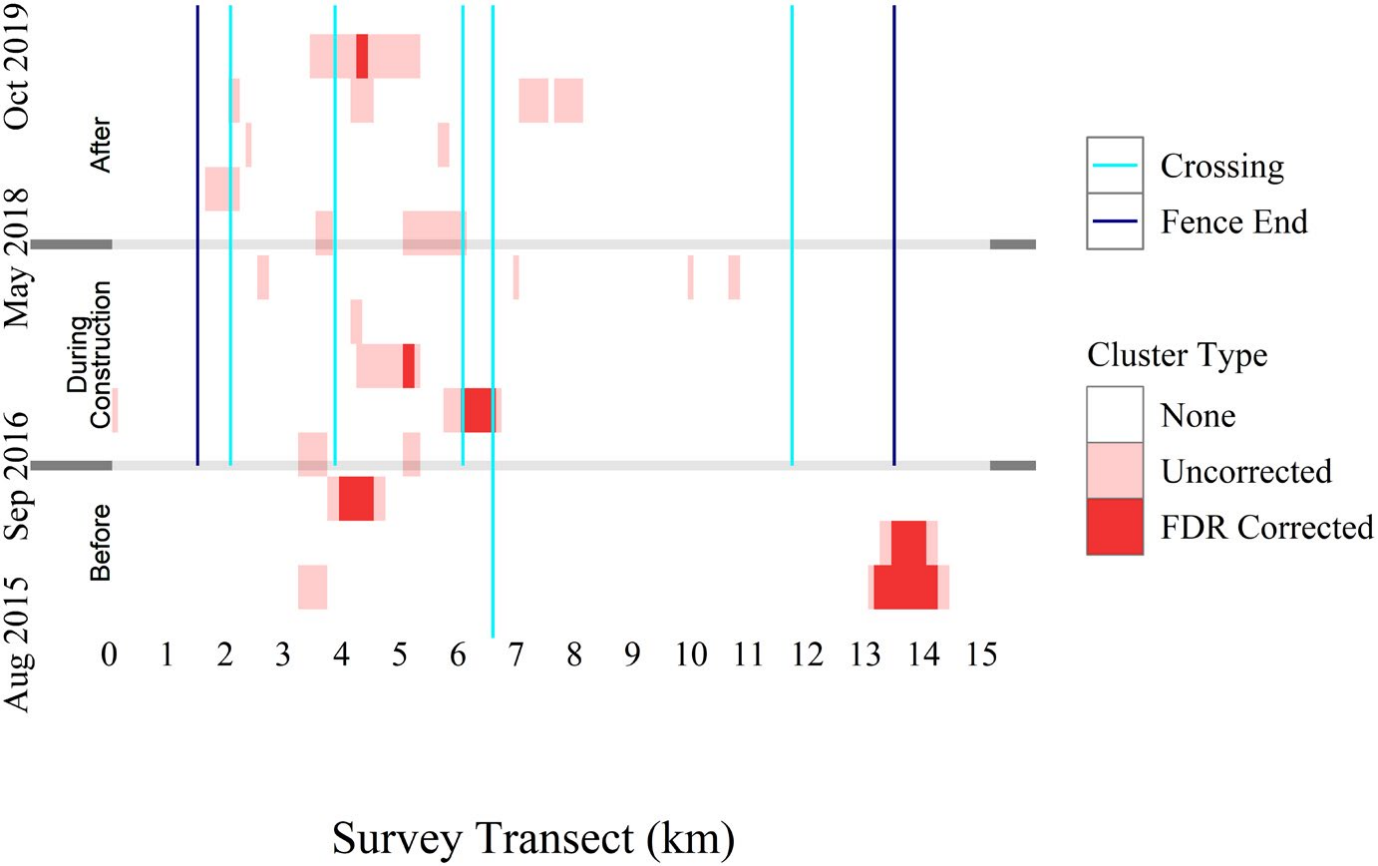
Heatmap of wildlife road mortality hot spots along State Highway 100, Cameron County, Texas. Statistically significant hot spots are those that were significant after applying the false discovery rate correction, while nonsignificant hot spots were those that were only significant without the correction. The survey transect blocks represent road segments and increase from west to east. To better relate this to the study area map, the approximate locations of wildlife crossings and fence ends are also indicated by vertical lines and the construction periods are indicated by horizontal lines

**FIGURE 6:**
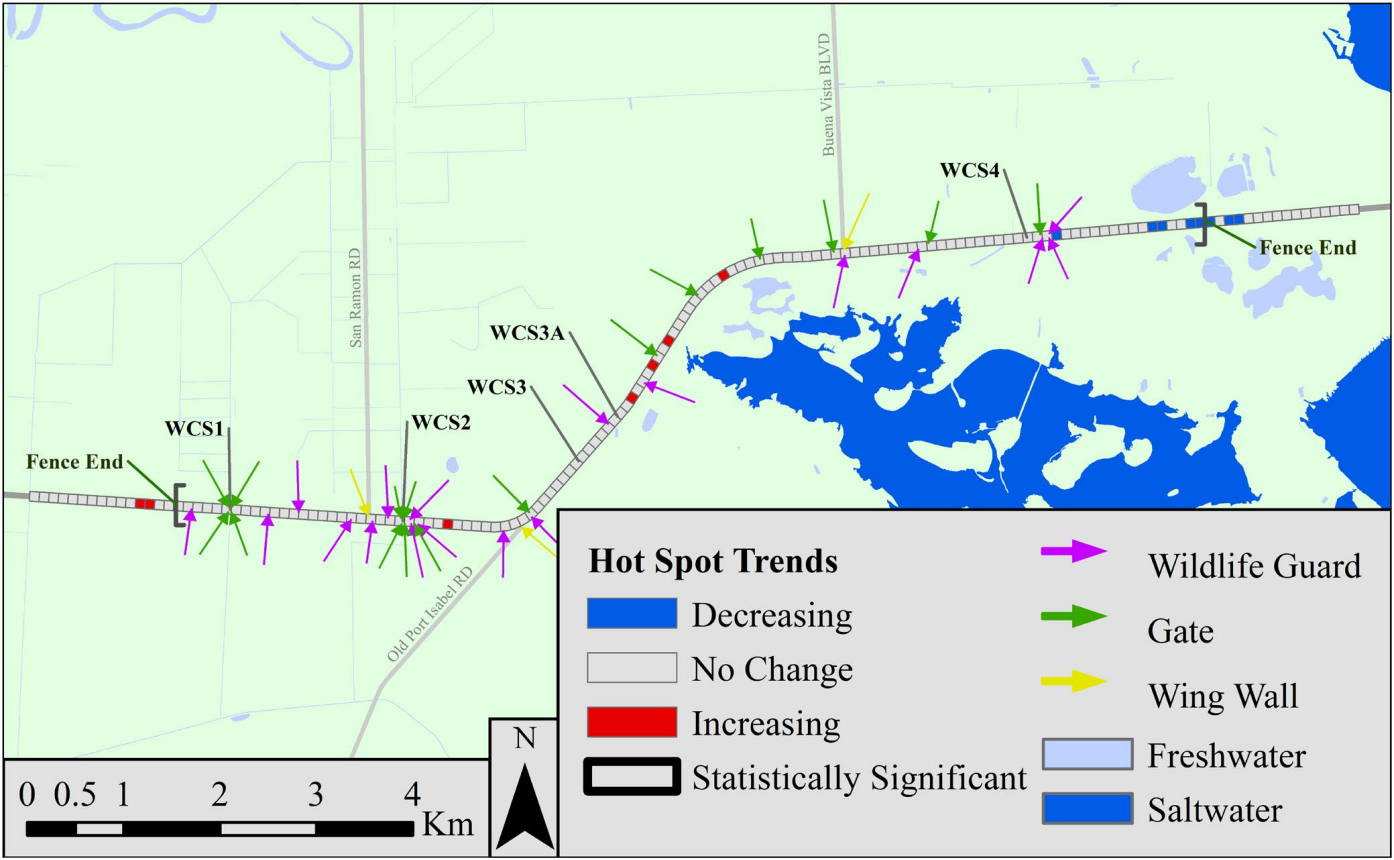
Trends in the intensity of wildlife road mortality (WRM) clusters along State Highway 100, Cameron County, Texas, from the Mann–Kendall trend test. Decreasing trends indicate that the intensity of WRM clusters decreased over time while increasing trends indicate that intensity of WRM clusters increased over time. No trends were statistically significant (at *α* = 0.05) after the false discovery rate (FDR) correction was applied

We identified hot spots in all time blocks, although the majority of these were not significant after applying the FDR correction (33 space time blocks out of a possible 1963 space time blocks; Figure 5). The majority of hot spots occurred on the western side of the survey transect and in similar locations as most of the WRMs. Additionally, the Mann–Kendall trend test revealed several increasing and decreasing trends in WRM hot spot intensity; however, none of these were statistically significant after applying the FDR correction (Figure 6).

### 3.2 Impacts of fence gaps on mortality trends

The PCA of distance to fence gaps indicated that approximately 85% of the variation among fence gap types was explained along the first PC axis (PC1), 8.0% on the second axis, 4.1% on the third, and 2.5% on the fourth (Figure 2). Distance to gates, wildlife guards, and wing walls were negatively correlated with PC1 (*r* = −0.96, −0.93, −0.93 respectively), and fencing was positively correlated with PC1 (*r* = 0.88; Figure 2).

Seven main effects and six interactions were included in the global model, giving a total of 793 possible models. The number of models included in the averaged model ranged from 3 (before construction) to 19 (during construction; Table 3). The range of McFadden pseudo‐*R* ^2^ values varied from 0.216–0.222 (after construction) to 0.329–0.333 (during construction). Six main effects and four interactions were included in the averaged model for the before construction period, all main effects and interactions were included in the averaged model for the during construction period, and six main effects and three interactions were included in the averaged model for the after construction period (Table 3).

**TABLE 3:** Summary of the averaged regression models for the effect of land cover and fence gaps on the intensity of wildlife road mortality clustering along State Highway 100, Cameron County, Texas

	Time period		
	Before	During	After
Models Included	3	19	6
(Intercept)	NS	−	−
Fence Gap Index	−	−	NS
Forested	NS	NS	−
Shrub	NS	+	+
Open	NS	+	NS
Agriculture	NS	NS	+
Developed	NS	NS	NS
Freshwater		NS	NS
Fence Gap × Forested		+	+
Fence Gap × Shrub	+	NS	−
Fence Gap × Open	+	NS	
Fence Gap × Agriculture	NS	NS	
Fence Gap × Developed	+	NS	NS
Fence Gap × Freshwater		NS	
Pseudo‐*R* ^2^ Range	0.248–0.255	0.329–0.333	0.216–0.222

Note

The factors included in the model were the distance to fence gaps principal components axis (Fence Gap Index), open vegetation (Open), shrubs (Shrub), forested, developed, agriculture, freshwater (Freshwater), and interactions between fence gap index and the land cover variables. The “models included” are the number of models used to compute the model‐averaged estimates and *p* values. Significance of a factor is indicated by a “+” (positive effect) or a “−” (negative effect). The pseudo‐*R* ^2^ range is the range of McFadden pseudo‐*R* ^2^ values for each model included in the averaged model.

Fence gap index had a significant negative relationship with intensity in the before construction period (slope = −1.50, *p* < .001) and during construction period (slope = −0.46, *p* < .001; Table 4). Forest proportion had a significant negative relationship with intensity in the after construction period (slope = −6.86, *p* < .001). Shrub proportion had a significant positive relationship with intensity in the during construction (slope = 6.52, *p* = .003) and after construction periods (slope = 12.63, *p* < .001). Open proportion had a significant positive relationship with intensity in the during construction period (slope = 2.35, *p* < .001). Agriculture proportion had a significant positive relationship with intensity in the after construction period (slope = 6.54, *p* < .01).

**TABLE 4:** Full model summaries for the averaged regression model assessing the effects on wildlife road mortality clustering on State Highway 100, Cameron County, Texas, for before, during, and after construction periods showing the estimated effect, standard error, Z score, and *p* value

Time period	Variable	Estimate	Adjusted SE	Z score	*p* value
Before	(Intercept)	−0.125	0.344	0.364	.716
	Fence Gap Index	−1.497	0.179	8.368	**.000**
	Agriculture	−5.492	11.900	0.462	.644
	Developed	−1.053	0.980	1.075	.282
	Open	−0.299	0.497	0.603	.547
	Shrub	0.124	1.546	0.080	0.936
	Fence Gap × Agriculture	10.924	11.470	0.952	.341
	Fence Gap × Developed	2.670	0.414	6.458	**.000**
	Fence Gap × Open	1.916	0.336	5.702	**.000**
	Fence Gap × Shrub	4.350	1.048	4.151	**.000**
	Forested	0.166	0.610	0.272	.786
During	(Intercept)	−1.931	0.311	6.208	**.000**
	Fence Gap Index	−0.456	0.117	3.894	**.000**
	Agriculture	−8.281	11.569	0.716	.474
	Forested	1.533	1.670	0.918	.359
	Open	2.345	0.504	4.652	**.000**
	Shrub	6.516	2.222	2.933	**.003**
	Fence Gap × Agriculture	14.936	11.149	1.340	.180
	Fence Gap × Forested	4.667	1.324	3.523	**.000**
	Fence Gap × Shrub	1.601	1.590	1.007	.314
	Developed	0.489	0.833	0.587	.557
	Fence Gap × Open	0.256	0.321	0.799	.424
	Freshwater	−3.687	11.073	0.333	0.739
	Fence Gap × Freshwater	1.921	10.058	0.191	.849
	Fence Gap × Developed	0.060	0.213	0.282	.778
After	(Intercept)	−0.721	0.345	2.091	**.037**
	Fence Gap Index	−0.201	0.179	1.124	.261
	Agriculture	6.540	1.244	5.259	**.000**
	Developed	0.421	0.871	0.484	.629
	Forested	−6.862	1.757	3.906	**.000**
	Freshwater	14.029	12.871	1.090	.276
	Open	−0.632	0.612	1.031	.303
	Shrub	12.632	2.091	6.040	**.000**
	Fence Gap × Developed	0.550	0.539	1.020	.308
	Fence Gap × Forested	5.100	1.390	3.669	**.000**
	Fence Gap × Shrub	−3.806	1.463	2.601	**.009**

Note

Significant effects are bolded.

In the before construction period, the relationship between fence gap index and intensity was affected by shrub proportion, open proportion, and developed proportion. At low levels of all three habitat variables, there was a strong, negative relationship between fence gap index and intensity, while at high proportions of the three habitat types, there was a weak positive relationship between fence gap index and intensity. In the during construction period, the relationship between fence gap index and intensity was strongly negative at low levels of forest proportion and strongly positive at high levels of forest proportion. In the after construction period, shrub proportion and forest proportion affected the relationship between fence gap index and intensity. At low levels of shrub proportion, there was a weak negative relationship while at high levels of shrub, there was a strong negative relationship between fence gap index and intensity. At low levels of forest proportion, there was a weak negative relationship between fence gap index and intensity, while at high levels of forest proportion, there was a strong positive relationship. Generally, relationships between intensity and habitat variables became weaker as fence gap index grew larger.

## 4 DISCUSSION

Overall, we found that at a fine temporal scale, the intensity of WRM clusters increased or decreased in few locations after construction of the mitigation structures on SH100, but none of these changes were significant after applying the FDR correction. Interestingly, the fence gap index showed a negative relationship with intensity in all three construction periods, although this effect was only significant in the before and during construction periods. Perhaps unsurprisingly, as forest proportion increased, WRM cluster intensity increased when closer to fence gaps in the during‐ and after construction periods. Generally, our two analyses agreed, indicating that, as of 1.5 years after construction of mitigation structures on SH100, WRM intensity has locally increased. However, although these locations were near fence gaps, they were not directly at fence gap locations. While intensity did increase in some locations, only one of these locations was associated with a statistically significant hot spot, indicating that WRMs are decreasing overall along SH100. Thus, with more time, we may expect to see additional decreasing trends in WRM clustering across most of the study area. Previous studies have shown that it may take years for wildlife to regularly use wildlife crossings (Clevenger & Waltho, 2005). Many of the wildlife crossings on SH100 occur near fence gaps, so as wildlife become familiar with wildlife crossings, we may see fewer animals attempting to cross on the road surface and fewer WRMs as a result.

We can draw several conclusions from these analyses. First, there appeared to be a geographical disparity between WRM clusters along the length of the transect. Second, when access to the highway is limited, habitat strongly affected how WRMs were related to distance to fence gaps. Finally, conducting local hot spot analysis at fine spatial and temporal scales can provide a unique picture of how WRM patterns change over time.

### 4.1 Wildlife road mortality distribution along SH100

As expected, WRMs/survey day decreased after construction of mitigation structures indicating that the mitigation structures are working to reduce WRMs on SH100. Most WRMs occurred on the western end of the survey transect, an area mostly consisting of agriculture and thornscrub habitat, with fewer WRMs occurring in areas with more open vegetation on the eastern side of the survey transect. One possible explanation for this is that there were fewer animals living around the eastern end of the survey transect. This area was made up primarily of oxeye daisy prairie, cordgrass prairie, and salt marsh (Elliott et al., 2014) which tended to have fewer species and fewer individuals than forested habitats in Cameron County (Yamashita, 2020). The western side of the transect was primarily agricultural and forested habitat, and both land cover types have been shown to be associated with greater WRM rates (Ascensão et al., 2017; Puglisi et al., 1974; Smith‐Patten & Patten, 2008). Therefore, while we could not measure this, it is possible that WRM rates may be similar along the length of the survey transect. It is also likely that wildlife living in disturbed habitats (such as those near agricultural lands) may be more willing to use road rights of way than individuals living in more natural habitats, thus increasing their risk of vehicle caused mortality (Forman et al., 2003).

In 2018 and 2019, there were favorable environmental conditions for population growth in many wildlife species in the study area, a factor that may have contributed to the limited changes seen in WRM clustering. Wildlife mitigation structures such as exclusionary fencing and wildlife crossings have been shown to increase wildlife populations living around roads (van der Ree et al., 2015), so the combination of favorable growth conditions and mitigation structures may have led to a decrease in the per capita WRM rate. Caceres (2011) showed that, in Brazil, abundance was the most significant predictor of WRM counts, so natural increases in animal abundance around SH100 may have led to increases in WRMs after construction. Therefore, limited changes seen in WRM cluster intensity may reflect increased wildlife populations rather than an ineffectiveness of mitigation structures. If wildlife populations are increasing around SH100 and wildlife crossings become more effective with time, then we would expect the decreasing trend in WRMs to continue.

Another contributing factor may be that there were more fence gaps on the western side of the survey transect than the eastern side. While this does not explain the high numbers of WRMs before or during construction, it may have contributed to the lack of decrease in WRMs seen after construction. The western side of the transect had 12 of 18 wildlife guards, 10 of 16 gates, and two of three wing walls offering multiple places for wildlife to access the road. The effects of different types of fence gaps were not examined in the present study, so it is possible that WRM cluster intensity may be higher around more permeable gaps such as wing walls or wildlife guards. Therefore, these mitigated fence gaps may not be as effective as gates at reducing wildlife access to the road.

### 4.2 Fence gaps and wildlife road mortality

Interestingly, our regression models indicated that WRM intensity increased with increasing distance to fence gaps across all three construction periods. However, we found statistically significant interactions with different habitat variables in all three construction periods which may have affected the identified relationship. Generally, WRM cluster intensity increased when nearer to (future) fence gaps when in areas with a high proportion of natural habitat (forested, shrubs, open), while intensity decreased when in areas of low natural habitat. Forested habitat had the strongest effect on the relationship between fence gaps, especially in the during‐ and after construction periods. Intensity of WRM clusters increased with increasing distance from fence gaps when forest proportion was low, and intensity increased with decreasing distance from fence gaps when forest proportion was high.

While we did document increases in WRM cluster intensity over time in some locations, we did not see evidence that fencing funneled animals onto SH100. Our documented locations of increased WRM cluster intensity did not occur at fence gaps; rather, they occurred 200–300 m from a gap. It is possible either that animals moved from fence gaps toward those locations while in the right of way before getting hit or that animals were climbing over or digging underneath the fence to get to the road at those locations. Cserkész et al. (2013) examined how WRM counts on a fenced highway were affected by distance to highway interchanges and demonstrated fencing funneled animals toward fence gaps. Fence gaps along SH100 occurred at high rates (3.1 gaps/km of highway) compared with the Cserkész et al. (2013) paper (0.12 gaps/km), thus creating more access points and diffusing WRMs across several kilometers of road instead of a single access point.

Our study indicated that there was limited change in WRM clustering with construction and that fence gaps were important, but not always significant, predictors of intensity in all three construction periods, thus indicating that fence gaps, especially in unforested areas, may be located in places previously used as wildlife travel corridors. In the after construction period, fence gaps probably represented known access points and likely had the highest chance of an animal crossing, similar to what McCollister and van Manen (2010) found after construction of wildlife mitigation structures in North Carolina, USA. Fence gaps represent a narrow access point, so assessing how they impact WRMs requires a local scale analysis (Červinka et al., 2015). At broader scales, the influence of access points to the highway may become masked by landscape‐level effects such as land cover and the presence of freshwater (Yamashita, 2020).

Finally, this study was conducted less than 2 years after the completion of mitigation structure construction, and it has been shown that wildlife may take several years to adjust to the presence of wildlife crossings (Clevenger, 2005; Clevenger & Waltho, 2005). It is possible that animals along SH100 were still in the “learning” phase and WRMs, especially around wildlife crossings, may begin to decrease as time passes. There is some visual evidence of this already with only three WRMs occurring within 200 m of four of the five wildlife crossings in the final two time blocks (8 months; Figure 4), indicating that animals may be preferentially using wildlife crossings instead of the roadway. However, it is unclear whether this was a result of learning or chance. Around one wildlife crossing (crossing 2), the large number of fence gaps near the crossing may increase the amount of time it takes wildlife to learn to use the crossing.

### 4.3 Using wildlife road mortality clusters to examine road mortality patterns

Using a location‐based clustering method to examine patterns of WRMs allowed us to determine the statistical significance of visually identified WRM hot spots. Knowing whether or not a cluster is significant can have important management implications because wildlife crossings can be expensive when they are built as a stand‐alone project (Huijser et al., 2009). Solely using counts of WRMs may miss important clustering of fewer WRMs which may benefit more from a wildlife crossing (Teixeira et al., 2017). The combination of hot spot analysis and time series analyses provides a framework for examining fine scale spatial and temporal patterns of WRMs, thus enabling assessment of how fine scale changes (i.e., wildlife mitigation structures) along a highway affect WRM patterns. This combination of hot spot and time series analyses can help determine how effective different mitigation structures are, an important question for managers and transportation agencies. Complementing this analysis with monitoring of wildlife mitigation structures using camera traps or another monitoring technique can allow managers to obtain a complete assessment of how wildlife mitigation structures benefit the animal community. Finally, hot spot analysis can provide useful visualizations of WRM data that can help display patterns hidden at larger scales. Generally, WRMs need to be examined at broad spatial and temporal scales due to sample size limitations. These analyses can miss important patterns occurring at finer scales (Levin, 1992). While local hot spot analysis likely has low power to detect changes in clustering due to low sample sizes in WRM datasets, it can provide useful representations of data that may elucidate previously unknown patterns in WRM datasets. For example, it would have been impossible to see that WRMs appeared to be declining around four of the wildlife crossings without the visualizations produced by this analysis.

While local hot spot analysis provides several benefits, the analysis requires large sample sizes to detect clusters so it is important to balance sample size limitations of the WRM dataset with the minimum spatial and temporal resolutions required for the local hot spot analysis and Mann–Kendall test (Caldas de Castro & Singer, 2006; Grubesic et al., 2014). For analysis purposes, medium to large mammal WRM rates tend to be fairly low (Ascensão et al., 2017). Therefore, the power of local hot spot analysis may be too low to detect significant WRM hot spots in medium to large mammals without very high WRMs or access to long‐term datasets. However, spatial indices of WRM rates, such as intensity of clusters, are essential to comparing long‐term WRM datasets where data collection and researcher experience may change over time. These sources of bias are likely to be consistent along an entire survey transect (Collinson et al., 2014), so they would not affect clustering patterns derived from WRM counts.

We assumed that WRM clustering was not affected by survey frequency and vehicle speed, but both sources of bias likely affected overall detections of WRMs and may have contributed to the reduced number of WRMs detected in the after construction period. By examining cluster intensity instead of WRM numbers, we focused on the relative distribution of WRMs through time and having fewer WRMs overall is unlikely to have a significant impact on hot spot intensity. It is possible that WRMs may be easier to detect along some parts of the survey transect when driving slower or that some areas may have lower carcass persistence times. Therefore, we believe that, because the locations of WRMs changed little through time (Figure 4), survey frequency and vehicle speed likely did not affect detection probability along different sections of the transect although more research is needed into how highway properties interact with vehicle speed and survey frequency to influence WRM detection probability.

The Mann–Kendall test requires a minimum of 10 time blocks to run (Harris et al., 2017; Hipel & Mcleod, 2005). To meet this requirement and maintain ecologically relevant time blocks, we divided WRMs into 4‐month time blocks. This meant that the total number of WRMs used to identify clustering for each time block (range 21–44) was likely too low to detect significant changes in clustering through time when applying a correction for multiple testing and spatial autocorrelation (Caldas de Castro & Singer, 2006; Grubesic et al., 2014). Therefore, an assessment of how sample size affects the power of local hot spot analysis will be required before this method can be applied more broadly.

## 5 CONCLUSIONS

We used local hot spot analysis, time series analysis, and generalized linear regression to examine how the construction of wildlife mitigation structures on SH100 affected the intensity of WRM clusters. While limited by the small sample sizes in each time block, our analysis provided a useful snapshot of how WRM spatial patterns change through time, so this technique should be limited to WRM datasets with long spatial and/or temporal scales. We recommend transportation managers conduct long‐term WRM surveys, especially in areas where mitigation structures such as wildlife crossings are employed to document whether WRMs are reduced.

By combining the local hot spot analysis, time series analyses, and regression, we demonstrated that the construction of exclusionary fencing and wildlife crossings reduced WRMs/survey day but did not significantly change spatial patterns of WRMs, possibly because fence gaps were located in places where WRM cluster intensity was high before construction. Visual inspection of fine scale WRM patterns, available from the local hot spot analyses, revealed that WRMs may be decreasing around wildlife crossings on SH100, indicating that the wildlife crossings were placed appropriately and animals may be learning that wildlife crossings provide a safer passageway across roads than the road surface. Additionally, fence gaps in forested areas may facilitate increased WRM cluster intensity, so reducing the number of gaps and mitigating necessary gaps with more effective structures, such as gates, will likely help reduce WRM rates. Therefore, local hot spot analysis, coupled with time series and regression techniques, can provide useful insights into how changes in the roadway impact wildlife use of the road area.

## CONFLICT OF INTEREST

None declared.

## AUTHOR CONTRIBUTIONS

**Thomas J. Yamashita:** Conceptualization (equal); data curation (equal); formal analysis (lead); investigation (equal); methodology (equal); visualization (lead); writing—original draft (lead); and writing—review and editing (equal). **Trinity D. Livingston:** Data curation (equal); investigation (equal); methodology (equal); and writing—review and editing (equal). **Kevin W. Ryer:** Conceptualization (equal); data curation (equal); formal analysis (supporting); investigation (equal); methodology (equal); project administration (supporting); visualization (supporting); and writing—review and editing (equal). **John H. Young:** Funding acquisition (equal); supervision (supporting); and writing—review and editing (equal). **Richard J. Kline:** Conceptualization (equal); funding acquisition (equal); project administration (lead); resources (lead); software (lead); supervision (lead); visualization (supporting); writing—original draft (supporting); and writing—review and editing (equal).

## Data Availability

Data used in this manuscript are available in the repository Dryad at https://doi.org/10.5061/dryad.qnk98sfhb

## APPENDIX A

Total number of Wildlife Road Mortalities on State Highway 100 by Species in the Before‐, During‐, and After Construction Periods

**TABLE A1:** Scientific names and counts of road mortalities in the before, during, and after construction periods for target species

Group	Class	Common name	Scientific name	Before	During	After	Total
Target Species	Mammalia	Virginia opossum	*Didelphis virginiana*	31	21	29	81
		Eastern cottontail	*Sylvilagus floridanus*	11	30	27	68
		Northern raccoon	*Procyon lotor*	18	30	12	60
		Coyote	*Canis latrans*	3	12	6	21
		Unknown skunk	*various*	0	8	9	17
		Domestic cat	*Felis catus*	2	7	8	17
		Nine‐banded armadillo	*Dasypus novemcinctus*	7	4	6	17
		Domestic dog	*Canis familiaris*	3	11	2	16
		Black‐tailed jackrabbit	*Lepus californicus*	4	8	3	15
		Unknown rabbit	*various*	0	2	8	10
		Unknown canid	*Canis* ssp.	5	0	0	5
		Bobcat	*Lynx rufus*	1	2	1	4
		Feral pig	*Sus scrofa*	0	2	0	2
		Javelina	*Pecari tajacu*	2	0	0	2
		White‐tailed deer	*Odocoileus virginianus*	0	2	0	2
		Nilgai	*Boselaphus tragocamelus*	0	1	1	2
		Striped skunk	*Mephitis mephitis*	0	0	2	2
		Unknown felid	*various*	1	0	0	1
		Nutria	*Myocastor coypus*	1	0	0	1
		Mammalia Total		89	140	114	343
	Reptilia	Texas tortoise	*Gopherus berlandieri*	8	4	5	17
		Red‐eared slider	*Trachemys scripta*	8	0	6	14
		Yellow mud turtle	*Kinosternon flavescnes*	5	0	5	10
		Unknown turtle	*various*	6	0	1	7
		Texas spiny softshell turtle	*Apalone spinifera*	1	0	0	1
		Reptilia Total		28	4	17	49
	Target Species Total			117	144	131	392

Note

Target species include most mammals, turtles, and tortoises.

**TABLE A2:** Scientific names and counts of mortalities by species in the before, during, and after construction periods for nontarget species

Group	Class	Common name	Scientific name	Before	During	After	Total
Nontarget Species	Aves	Unknown bird	*various*	1	22	47	70
		Barn owl	*Tyto alba*	0	7	12	19
		Black‐bellied whistling duck	*Dednrocygna autumnalis*	0	0	11	11
		Eastern meadowlark	*Sturnella magna*	0	1	8	9
		Northern mockingbird	*Mimus polyglottos*	0	1	5	6
		Northern bobwhite	*Colinus virginianus*	1	3	1	5
		American coot	*Fulica americana*	0	5	0	5
		Unknown duck	*various*	2	0	1	3
		Osprey	*Pandion haliaetus*	0	1	1	2
		Long‐billed thrasher	*Toxostoma longirostre*	0	0	2	2
		Greater roadrunner	*Geococcyx californianus*	0	1	1	2
		Seagull	*various*	0	2	0	2
		Green heron	*Butorides virescens*	1	0	1	2
		Western kingbird	*Tyrannus verticalis*	0	1	1	2
		Yellow‐billed cuckoo	*Coccyzus americanus*	0	1	1	2
		Night hawk	*Chordeiles* ssp.	0	1	1	2
		Killdeer	*Charadrius vociferus*	0	1	0	1
		Black vulture	*Coragyps atratus*	0	0	1	1
		Unknown goose	*various*	0	0	1	1
		Common pauraque	*Nyctidromus albicollis*	0	0	1	1
		Harris hawk	*Parabuteo unicinctus*	0	1	0	1
		Northern cardinal	*Cardinalis cardinalis*	0	0	1	1
		Unknown bird‐ small	*various*	0	0	1	1
		Common starling	*Sturnus vulgaris*	0	0	1	1
		Mourning dove	*Zenaida macroura*	0	0	1	1
		Laughing gull	*Leucophaeus atricilla*	0	0	1	1
		Rock dove	*Columba livia*	0	1	0	1
		Loggerhead shrike	*Lanius ludovicianus*	0	1	0	1
		Aves Total		5	50	101	156
Nontarget Species	Mammalia	Unknown mammal	*various*	20	7	5	32
		Unknown rat	*various*	2	5	13	20
		Unknown rodent	*various*	0	0	5	5
		Muridae rat	*various*	5	0	0	5
		Long‐tailed weasel	*Mustela frenata*	4	0	1	5
		Cotton rat	*Sigmodon* ssp.	2	0	0	2
		Cricetidae rat	*various*	2	0	0	2
		Hispid cotton rat	*Sigmodon hispidus*	1	0	0	1
		Mexican ground squirrel	*Spermophilus mexicanus*	0	0	1	1
		Mammalia Total		36	12	25	73
	Reptilia	Western diamondback rattlesnake	*Crotalus atrox*	22	12	13	47
		Unknown snake	*various*	20	5	15	40
		Great plains rat snake	*Elaphe emoryi*	12	0	2	14
		Western ribbon snake	*Thamnophis proximus*	7	0	3	10
		Texas indigo snake	*Drymarchon melanurus erebennus*	1	0	3	4
		Western coachwhip	*Masticophis flagellum testaceus*	1	0	2	3
		Mexican racer	*Coluber constrictor oaxaca*	2	1	0	3
		Rat snake	*Pantherophis* ssp.	0	1	0	1
		Unknown reptile	*various*	1	0	0	1
		Masticophis ssp.	*Masticophis* ssp.	0	0	1	1
		Texas patchnose snake	*Salvadora grahamiae*	1	0	0	1
		Reptilia Total		67	19	39	125
	Amphibia	Amphibia Total		0	0	13	13
	Malacostraca	Malacostraca Total		0	0	6	6
	Unknown	Unknown Total		1	0	1	2
	Nontarget Species Total			109	81	185	375
**Grand Total**				**226**	**225**	**316**	**767**

Note

These primarily included birds, small mammals, and snakes.
